# Clinical letters to patients with intellectual disabilities after psychiatric review: A quality improvement project

**DOI:** 10.1177/17446295211046478

**Published:** 2021-12-22

**Authors:** Indermeet Sawhney, Asif Zia, Bob Gates, Anu Sharma, Adetayo Adeniji

**Affiliations:** Hertfordshire Partnership University NHS Foundation Trust, UK; Hertfordshire Partnership University NHS Foundation Trust, UK; University of West London, UK; University of West London, UK; University of Hertfordshire, UK; University of Derby, UK; Hertfordshire Partnership University NHS Foundation Trust, UK; Hertfordshire Partnership University NHS Foundation Trust, UK

**Keywords:** intellectual disabilities, psychiatric review, quality improvement project, clinical letters

## Abstract

**Aim:**

This Quality Improvement Project sought to improve communication between patients with intellectual disabilities and their psychiatrists by sharing medical information using an easy read letter format following psychiatric review.

**Background:**

Writing directly to patients is in keeping with good medical practice. Previous studies have shown patients with intellectual disabilities prefer letters tailored to meet their needs.

**Method:**

An easy read letter was used by nine psychiatrists who handed them to 100 consecutive patients after review. Feedback of acceptability to patients was obtained using a three-item facial rating scale and the use of free text. Feedback of acceptability was obtained from participating psychiatrists.

**Results:**

Patients found the easy read letter helpful and felt it should be used routinely. Psychiatrists felt this approach was beneficial as well as aiding patient understanding of review.

**Conclusions:**

The easy read letter was reported to improve communication following psychiatric review. Limitations are acknowledged but it is concluded that an easy read letter should be adopted as routine practice following psychiatric review, for people with intellectual disabilities.

## Background

In accordance with the National Health Service (NHS) Constitution (2021), patients in the United Kingdom (UK) have a right to be involved both in the planning and with the making of decisions about their health and care by their care providers. They also have a right to be given information and support to enable them to do this, and where appropriate, this right extends to their family and carers. Additionally, since the publication of the NHS Plan patients have the right to be copied into letters written about them from one health professional to another (Great Britain, Department of Health, 2000). The overriding objectives of the development of such rights was to improve communication with patients, and to enable them to participate in their care. Nevertheless, current clinical practice in the UK is for hospital doctors to write letters to patients’ General Practitioners (GPs) following an outpatient consultation, and for patients to receive a copy of this.

The Equality Act 2010 placed a legal duty on all service providers to take steps or make ‘*reasonable adjustments*’ to avoid placing a disabled person at a substantial disadvantage when compared to persons who are not disabled. This Act is explicit in its requirement for including the provision of information in ‘*an accessible format*’ as a ‘*reasonable step*’ to be taken. Furthermore, all organizations that provide NHS care and, or, publicly funded adult social care are legally required to follow the Accessible Information Standard, which came into force in 2016. The standards set out a specific, and consistent approach to meeting the information and communication support needs of patients, service users, carers and parents with disabilities, impairment, or sensory loss. These standards place a statutory obligation on organizations to ensure people receive information in accessible formats, which they are able comprehend (NHS, 2016).

Additionally, the General Medical Council, the professional body and regulator for doctors in the UK, in its *Good Medical Practice* guidance for doctors advises medical practitioners state that, *they must give patients information they want, or need to know, in a way that they can understand* (General Medical Council, 2021). To assist medical doctors, in this respect, the Academy of Medical Royal Colleges (AoMRC) has published guidance to ensure that they meet this requirement. This guidance encourages medical doctors to write their outpatient clinic letters directly to patients rather than simply copying them into letters to the patients GP, and to use understandable terminology rather than the more commonly and often used complex medical jargon (Academy of Royal Medical Colleges, 2018).

One study has shown that professionals working in intellectual disability services, believe that a simplified version of the letter sent to the GP, following psychiatric review, should be given to patients with intellectual disabilities making it more meaningful for this patient group (Sawhney et al., 2007). This study also found that patients with intellectual disabilities indicated that they had a tendency to forget what is discussed in a psychiatric review and felt their understanding of mental illness could be increased by receiving letters given to them directly. This study also noted that patients with intellectual disabilities have shown a preference to receive a separate simple letter in a large font (Sawhney et al., 2007).

## Aims

This QIP^1^ describes a simple, yet innovative *reasonable adjustment* to current clinical medical practice in England, UK to ensure patients with intellectual disabilities were written to directly in an accessible format by their psychiatrists after psychiatric review. The aims of the project were twofold. Firstly, it sought to ascertain views, experience and acceptability to people with intellectual disabilities and their carers as to the use of a review letter presented in an accessible format. Secondly it sought the views of, and acceptability to the participating psychiatrists about writing to patients using such an approach.

## Participants and setting

A purposeful non-probability convenience sample of patients, (N = 61 male, 39 females, with a mean age 42), were consecutively recruited into this QIP until N = 100 was reached; this being the agreed number for the QIP, and within the resource available to support the project and within ongoing clinical practice. Recruitment was undertaken across the existing clinical caseloads of the nine participating psychiatrists. Of the patients participating N = 35 had mild, 38 moderate, and 27 severe intellectual disabilities. All patients had a diagnosis of one or more mental illness and, or epilepsy as a co-morbidity to their intellectual disabilities. They presented with: Mood Disorder N = 34, Psychosis (Schizophrenia and /Schizoaffective Disorder) N = 7, Neurodevelopmental Disorders N = 35, Epilepsy N = 48 and Anxiety Disorder N = 14. All patients were prescribed psychotropic and, or anti-epileptic medication. They were prescribed several medicines: Antipsychotic N = 50, Antidepressants N = 28, Antiepileptic N = 64, Stimulants N = 3 and Mood stabilizers N = 4. All the psychiatrists were specialists and specialized in working with and supporting people with intellectual disabilities. All participating patients and psychiatrists originated from two counties in England, Hertfordshire and Essex, over a period of 3 months.

## Ethical issues

The Hertfordshire Partnership University NHS Foundation Trust identified that as the proposal was for a QIP it should be classified as a service evaluation, and as such, its remit fell outside the governance arrangements of NHS research committees; and it was registered as such with the host NHS organization as a QIP. Regardless of governance arrangements, the project was conducted within the general ethical conventions of social research (Haber, 1998) and was conducted in a manner which respected those participating in the project, and was additionally concerned for their dignity and welfare, as set out in the Research Governance Framework for Health and Social Care (DH, 2005). No data with shared outside of the clinical team, and no member of the team had any identifiable patient information, other than their own patients.

## Method

A standard ‘*easy read*’ clinic letter template was developed through co-design by clinicians with input from experts by experience comprising, families, carers and people with intellectual disabilities, to be sent by psychiatrists after psychiatric review to patients following their attendance at an outpatient clinic. Self-explanatory pictures and symbols depicting different facets of health and care were incorporated into the letter to facilitate understanding by people with intellectual disabilities. The easy read letter was handed to each patient /carer at the end of their appointment, at either the outpatient clinic appointment or home visit, following their psychiatric review.

**Figure 1.**
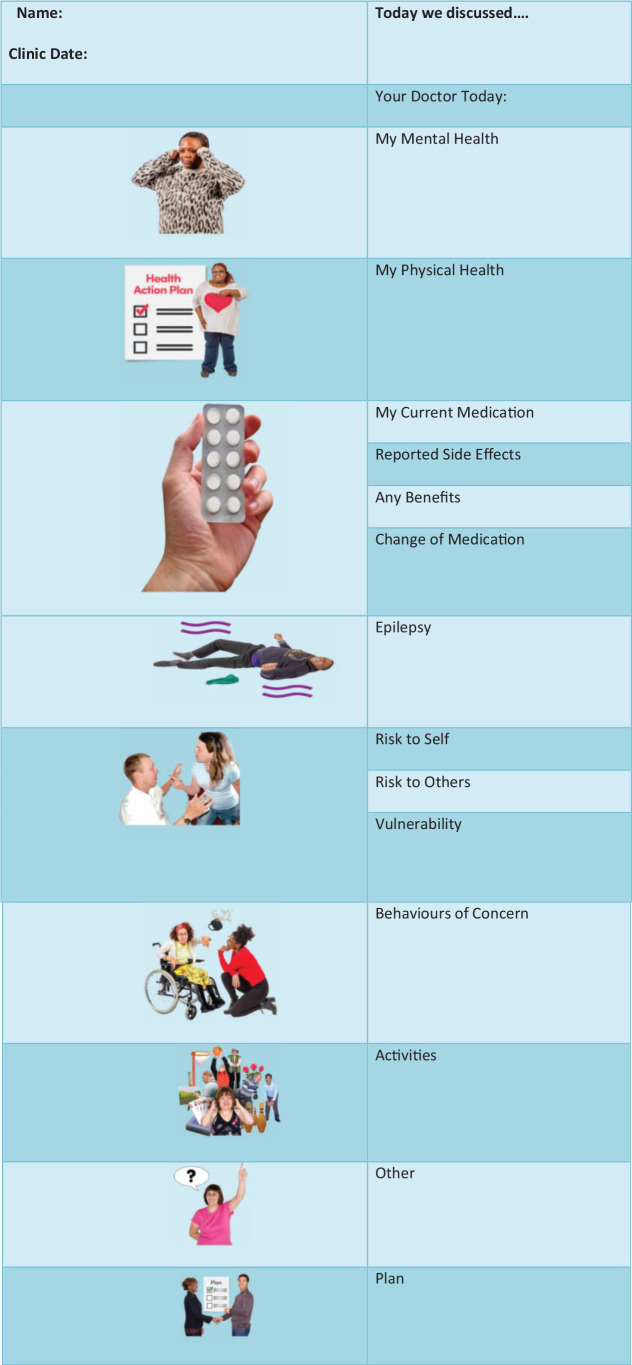
Appointment outcome letter.

The easy read letter began with an introduction by the doctor (with photograph) who undertook the review, and it then incorporated several separate subheadings which covered; mental health, physical health, current medication (and the benefits and side effects if any) and changes to, medication. It also included epilepsy, risks (risks to self and to others), vulnerability, behaviours of concern and day-to-day activities, and finally a heading about a plan which was formulated at the end of the consultation (see Figure 1). This proforma did not replace the routine clinical letter which was sent to the GP following the existing protocol.

**Figure 2.**
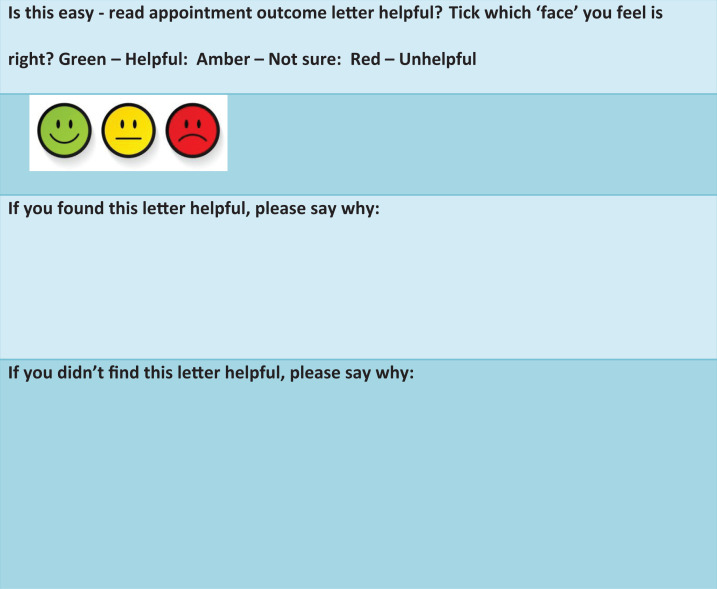
Facial three-item Likert rating scale and free text boxes.

A second ‘*easy read*’ facial recognition Likert type scale was designed to ascertain their satisfaction, or otherwise, of the format. In addition, free text boxes were also provided on this form to capture the views and experiences of patients receiving such letters. Patients and, or carers were asked to rate the letter using the three-point Likert scale – Green for ‘*helpful*’, Amber for ‘*not sure*’ and Red for ‘*not helpful*’; the use of such scales and in novel ways is now common practice in health-related research such as: medicine, nursing and psychology (McLeod, 2019). They were then asked if they found the format of the letter helpful, or not, and were asked to give reasons for their answers in the text boxes provided (see Figure 2).

Additionally, feedback was sought and obtained from the psychiatrists participating in the QIP regarding their experience and the acceptability of incorporating the easy read template in their psychiatric clinics. Post clinic all data sources from both patients and the psychiatrists were collected and analysed by members of the QIP team.

## Findings

There were unanimous and positive responses from all the participating patients; with 100% of respondents indicating that they found the easy read letter helpful. Whereas not all patients or carers could, or chose to, provide written textual comments on why they found the letter helpful, they all ticked the green ‘*smiley*’ face, indicating a positive experience. It was not always possible to differentiate between the respondents making comments on the form. Some carers helped patients complete the form, patients sometimes directed carers, while other patients completed it themselves. It is known that eight patients and, or their carers added textual comments to the form. There were no negative comments from patients or carers regarding their experience. Some of the text comments that were proffered by eight patients/carers identified why they found the format of this easy read letter helpful:

‘*good reminder on how the appointment went*’, [Carer or Patient]
A reminder of ‘*what was discussed*’, [Patient]
‘*easy to read, easier to understand with pictures*’, [Patient]
‘*improved understanding*’, [Patient]
‘*breakdown of headings made it easy*’, [Patient]
‘*clear and concise information*’, [Carer or Patient]
‘*helped staff supporting remember what happened in the appointment*’, [Carer]
‘*good reflection of what was discussed*’. [Carer]

The feedback received from the nine participating psychiatrists in this QIP indicated they felt that the initiative improved patient understanding, and thereby improved the therapeutic relationship between themselves as clinician and their patients. Other comments received from N = 3 psychiatrists included its potential to:

‘*empower patients, become engaged in their care*’, and [Psych],
‘*enable them to make decisions about their treatment plans*’ as well as, [Psych],
‘*leading to greater patient satisfaction*’ [Psych].

However, concerns were raised by some clinicians regarding the use of the form as being a time-consuming exercise which could impact on their workload.

## Discussion

Outpatient correspondence to GPs are probably the most frequently written letters in the NHS in the UK. Such letters communicate clinical information and management plans made by specialist medical practitioners of patients to their GP. Additionally, such correspondence serves to form and inform part of a patient’s clinical record, and as such they are an important reference point as to what was discussed at clinic. Furthermore, patients can alert the wider network of clinicians who support them of errors and or update them as to changes made to their treatment plan at an outpatient clinic, thus enabling a more co-ordinated care approach.

Within the UK, current clinical practice is for hospital doctors to write such letters to the patients’ GP following an outpatient consultation, and for these patients to receive a copy of the letter. However, new guidance now advises doctors to write directly to patients (Academy of Royal Medical Colleges, 2018). And where doctors have adopted the practice of writing directly to patients, evidence suggest that the communication style becomes more ‘*patient centred*’ (Logan et al., 2019). Studies have also shown that GPs are supportive of the practice of writing directly to patients, as it improves understanding of their patients resulting in GPs spending less time interpreting meaning of the content to patients (Academy of Royal Medical Colleges, 2018). The benefits of writing directly to patients has now been established in various studies, and in different branches of medicine for the wider population (Logan et al., 2019; Lonergan et al., 2019). Most importantly, studies show that patients find such letters useful, supportive, and informative, and this is in keeping with the findings of this project. Studies have also revealed that patients find such letters an aide memoire of what was discussed during their consultation – complex issues can be easily forgotten (Academy of Royal Medical Colleges, 2018). This latter point is particularly relevant for people with intellectual disabilities who may be more likely to forget due to their cognitive impairments. Finally, some studies have demonstrated that once patients who received such letters directly wished for this practice to be continued (Baker et al., 2002).

However, there seems to be less agreement around the comprehension of the content of the letters (Roberts and Partridge, 2006). In this QIP the review letter was developed and co-designed with people with intellectual disabilities as an easy read template to promote comprehension for these patients. To make it meaningful it was paramount that both the language used, and the information provided is constructed to be congruent to a patient’s level of understanding. To achieve this, it is likely adaptations such as use of pictures or diagrams to facilitate better understanding as was undertaken in this QIP will need to be incorporated. It is suggested that it is an imperative for specialist clinicians to communicate directly with the people with intellectual disabilities, although such correspondence to the patient is not intended to replace clinic outcome letters that are routinely forwarded to the GP and other professionals involved in the care of the patient.

Whereas there was merit in the concerns expressed by these psychiatrists that their workload could potentially increase by sending such letters patients it is suggested that over time doctors would get used to and accommodate writing to patients in this way, and this would inevitably speed up the process. Moreover, and perhaps more importantly communicating effectively with patients is known to be central to being a good doctor, and such practices need to be incorporated into routine clinical practice, and nowhere is this more important than in specialist intellectual disability services that should act as a beacon of exemplar practice in making reasonable adjustments. This is especially relevant as writing directly to patients, in an accessible way, rather than sending them copy of the letter sent to the GP is known to improve communication with patients with intellectual disabilities. Direct communication with people with intellectual disabilities in an accessible fashion helps to empower them and is an important move towards treating them as equal partners in a consultation. Additionally, patients with intellectual disabilities will have a written record of the consultation which they can share with others involved in their care, thus ensuring more coordinated care. Also, having information to hand in an accessible manner will place them in a better position to be able to take in the information and advice given by the doctor. This is important because in enabling people with intellectual disabilities to have more control of their lives and make better choices about their health is dependent on them understanding the information imparted to them at such reviews. Furthermore, this practice should not be confined to medical but should and could be adopted by all professionals in specialist intellectual disability services and teams to enhance care and support for people with intellectual disabilities. As with all research, evaluations of clinical practice and in this case QIPs potential limitations and weaknesses are inevitable. One such limitation is the potential of acquiescence of people with intellectual disabilities in the research process. A long-standing assumption since the 1980s has been that the involvement of people with intellectual disabilities compromises the results of any intervention or research because of ‘*acquiescence bias*’. Such a view had become commonplace in the research literature in the field of intellectual disabilities (Sigelman et al., 1981). This relatively commonplace wisdom has endured for some time and has now been challenged more recently by Rapley and Antaki (1996) who have said:

‘acquiescence bias’ in the responses of people with learning disabilities to questioning is not a simple phenomenon, and certainly not one to be laid at the door solely of people with learning disabilities themselves. Rather, it is probably an artefact of the conversational organization of interviews as tests. (Rapley and Antaki, 1996: 207–227)

We concur with Rapley and Antaki (1996) and further assert that this portrayal of a group of people as submissive and presenting with ‘*willing-to-please acquiescence*’ is neither sustainable as a research assumption, or grounded in any convincing evidence. Perhaps arguably in more enlightened times this persistent belief of acquiescence should be replaced with new more inclusive thinking, and by a more courteous and respectful account of the abilities of people with intellectual disabilities. That said judicious caution in our interpretation of data is advised, and we see the failure to ‘capture’ the origins of the source of the textual data as a weakness. The team might also have been more circumspect in relation to the collection of the data in relation to level of [dis]ability, diagnosis and treatment regimen, and controlling for the effects on these variables; this might have offered other clinicians more confidence in the findings and our interpretation of these data in this QIP.

## Conclusion

This Quality Improvement Project has described the implementation of an intervention to improve the quality of outpatient letters as to their accessibility, acceptability, and value to patients with intellectual disabilities following psychiatric review. It is concluded that specialist intellectual disability services need to ensure that outpatient psychiatric review information is imparted to patients and, where appropriate their carers, in an accessible manner. On balance we believe that writing to patients using an easy read template as, demonstrated from this project, should be incorporated and considered as standard clinical practice. The adoption of such practice would address potential shortcomings in contemporary clinical medical practice by addressing the standards required in the Equality Act 2011, and Good Medical Practice (General Medical Council, 2021). As importantly as demonstrated in this QIP patient, carer and psychiatrists’ feedback indicate that these letters improved overall understanding at the point of post psychiatric clinic consultation, and that people with intellectual disabilities found them to be helpful.

## Note

1. Quality Improvement Projects are used widely in the National Health Service in the UK to introduce changes to clinical practice that might lead to better health outcomes care, and, or performance by health care professionals.

## References

[bibr1-17446295211046478] Academy of Royal Medical Colleges (2018) Please, write to me. Writing outpatient clinic letters to patients – guidance. Available at: https://www.aomrc.org.uk/reports-guidance/please-write-to-me-writing-outpatient-clinic-letters-to-patients-guidance/ (accessed 1 March 2021).

[bibr2-17446295211046478] Baker DL Eash T Schuette JL, et al. (2002) Guidelines for writing letters to patients. Journal of Genetic Counselling 1: 399–418. DOI: 10.1023/A:1016841731426. 10.1023/A:1016841731426 26142130

[bibr3-17446295211046478] Department of Health (2005) Research Governance Framework for Health and Social Care, 2nd edn. London: The Stationery Office.

[bibr4-17446295211046478] Equality Act (2010) The Stationary Office. London. Available at: www.legislation.gov.uk/ukpga/2010/15/contents (accessed 1 March 2021).

[bibr5-17446295211046478] General Medical Council (2021) The duties of a doctor registered with the General Medical Council. Available at: https://www.gmc-uk.org/ethical-guidance/ethical-guidance-for-doctors/good-medical-practice/duties-of-a-doctor.31-34.(accessed 1 March 2021). 11895257

[bibr6-17446295211046478] Great Britain, Department of Health (2000) The NHS Plan: A Plan for Investment, A Plan for Reform. Norwich: The Stationary Office (Cm. 4818-1).

[bibr7-17446295211046478] Haber J (1998) Legal and ethical issues. In: LoBiondo-Wood G Haber J (eds) Nursing Research: Methods, Critical Appraisal and Utilization, 4th edn. St. Louis, MO: Mosby-Year Book Inc, Chapter 11, pp. 232–246.

[bibr8-17446295211046478] Logan I Bestford G Tomson C, et al. (2019) Benefits to the nephrology MDT of writing outpatient letters directly to patients. UK Kidney Week. Available at: http://www.ukkw.org.uk/wp-content/uploads/2019/08/P448.pdf (accessed 1 March 2021).

[bibr9-17446295211046478] Lonergan PE Gnanappiragasam S Redmond EJ, et al. (2019) Write2me: using patient feedback to improve post consultation urology clinic letters. British Medical Journal Open Quality 8: e000721. Available at: https://bmjopenquality.bmj.com/content/8/3/e000721 (accessed 1 March 2021). 10.1136/bmjoq-2019-000721 PMC6768383 31637326

[bibr10-17446295211046478] McLeod SA (2019) Likert scale. Simply Psychology. Available at: https://www.simplypsychology.org/likert-scale.html (accessed 1 March 2021).

[bibr11-17446295211046478] National Health Service (2016) Accessible information standard: making health and social care information accessible. Available at: https://www.england.nhs.uk/ourwork/accessibleinfo/ (accessed 1 March 2021).

[bibr12-17446295211046478] National Health Service (2021) National Health Service Constitution. Available at: https://www.gov.uk/government/publications/the-nhs-constitution-for-england/the-nhs-constitution-for-england (accessed 1 March 2021).

[bibr13-17446295211046478] Rapley M Antakii C (1996) A conversation analysis of the ‘acquiescence’ of people with learning disabilities. Journal of Community & Applied Social Psychology 6(3): 207–227.

[bibr14-17446295211046478] Roberts NJ Partridge MR (2006) How useful are post consultation letters to patients? BMC Medicine 4: 2. 16426444 10.1186/1741-7015-4-2 PMC1360088

[bibr15-17446295211046478] Sawhney I Morgan J Tajer A (2007) Copying letters to patients: views of professionals working with people with learning disability. Tizard Learning Disability Review 12(4): 42–48.

[bibr16-17446295211046478] Sigelman CK Budd EC Spanhel CL, et al. (1981) When in doubt, say yes: acquiescence in interviews with mentally retarded persons. Mental Retardation 19: 53–58. 7231176

